# Changes in preterm birth and birthweight during the SARS-CoV-2 pandemic: a nationwide study in South Korea

**DOI:** 10.1038/s41598-022-20049-2

**Published:** 2022-09-29

**Authors:** Jeongeun Hwang, Seokjoo Moon, Kyu-Dong Cho, Min-Jeong Oh, Su Jung Hong, Geum Joon Cho

**Affiliations:** 1 Division of Medical Oncology, Department of Internal Medicine, Korea University College of Medicine, Seoul, Republic of Korea; 2 Department of Biomedical Research Center, Korea University Guro Hospital, Seoul, Republic of Korea; 3 Smart Healthcare Center, Korea University Guro Hospital, Seoul, Republic of Korea; 4 Big Data Department, National Health Insurance Service, Gangwon-do, Republic of Korea; 5 Department of Obstetrics and Gynecology, Korea University College of Medicine, Seoul, Republic of Korea

**Keywords:** Paediatrics, Public health, Paediatric research

## Abstract

Birthweight is a strong determinant of a neonate’s health. The SARS-CoV-2 pandemic’s impact on birthweight has not been investigated in-depth, with inconsistent conclusions from initial studies. To assess changes in preterm birth and inappropriate birthweight between the SARS-CoV-2 pandemic and pre-pandemic periods. A nationwide birth micro-data consisted with exhaustive census of all births in 2011–2020 in South Korea was accessed to examine whether the mean birthweight and rates of under/overweight births changed significantly during the SARS-CoV-2 pandemic year (2020) compared to those of the pre-pandemic period (2011–2019). A total of 3,736,447 singleton births were analyzed. Preterm birth was defined as < 37 weeks of gestation. Low birthweight (LBW) and macrosomia were defined as birthweights < 2.5 kg and ≥ 4.0 kg, respectively. Small for gestational age (SGA) and large for gestational age (LGA) were defined as birthweights below the 10th and above 90th percentiles for sex and gestational age, respectively. Inappropriate birthweight was defined as one or more LBW, macrosomia, SGA, or LGA. Generalized linear models predicted birth outcomes and were adjusted for parental age and education level, marital status, parity, gestational age, and months from January 2011. There were 3,481,423 and 255,024 singleton births during the pre-pandemic and pandemic periods, respectively. Multivariable generalized linear models estimated negative associations between the pandemic and preterm birth (odds ratio [OR], 0.968; 95% confidence interval [CI] 0.958–0.978), LBW (OR: 0.967, 95% CI 0.956–0.979), macrosomia (OR: 0.899, 95% CI 0.886–0.912), SGA (OR: 0.974, 95% CI 0.964–0.983), LGA (OR: 0.952, 95% CI 0.945–0.959), and inappropriate birthweight (OR: 0.958, 95% CI 0.952–0.963), indicating a decline during the pandemic compared to pre-pandemic period. An 8.98 g decrease in birthweight (95% CI 7.98–9.99) was estimated during the pandemic. This is the largest and comprehensive nationwide study to date on the impact of the SARS-CoV-2 pandemic on preterm birth and inappropriate birthweight. Birth during the pandemic was associated with lower odds of being preterm, underweight, and overweight. Further studies are required to understand the dynamics underlying this phenomenon.

## Introduction

Pregnant women are at a higher risk of complications associated with SARS-CoV-2, such as ICU admission and death, and SARS-CoV-2 infection during pregnancy increases the risk of adverse birth outcomes, including preeclampsia, preterm birth, and stillbirth^1–3^. In addition to the direct impact of SARS-CoV-2 infection on pregnancy outcomes, the pandemic and ensuing government response have had possible adverse effects on pregnancy outcomes, even among those not infected by SARS-CoV-2. Increases in stillbirths and maternal deaths^4^ and, paradoxically, an overall decline in preterm births^5–9^, or little change in preterm births^10–12^ were observed during the pandemic compared to before the pandemic.

Birthweight is a strong determinant of infant’s health. Babies born with inappropriate birthweight are at high risk of developing increased risk for perinatal morbidity and long-term health complications^13–17^. The SARS-CoV-2 pandemic has profoundly changed the lifestyle, physical and mental health, and health care access^18–21^ of pregnant women; these are known stressors affecting fetal growth^22–26^. However, the cumulative impact of these stressors aggravated by the SARS-CoV-2 pandemic on fetal growth and birthweight has not been examined in detail, and initial evidence has been inconsistent^8–10,21,27^.

The characteristics of the datasets varied among the aforementioned studies concerning the impact of the SARS-CoV-2 pandemic on preterm birth or birthweight. There were studies based on registry datasets^5–7,12,21^, claims data of a commercial insurance network^10^, single hospital^8,27^, or 2 hospitals^11^ data. A study by Wagner et al.^21^ analyzed nationwide birth registry data in Austria on adverse birth outcomes and birthweight, but did not report on preterm birth. Studies by Been et al.^5^, Oakley et al.^12^, and Yalcin et al.^9^ analyzed national registry datasets including more than a million births each to investigate preterm birth but neither studied macrosomia. A study by Sun et al.^10^ was interested in both preterm birth and birthweight, but they used claims data of a commercial insurance network thereby limiting their generalizability to pregnancy outside the United States commercial insurance system. Kim et al.^8^ studied both preterm birth and low birthweight before and after the pandemic but in a single hospital.

The objective of this study was to evaluate the effects of the SARS-CoV-2 pandemic on preterm birth and birthweight and to add one of the largest and the most comprehensive pieces of evidence by analyzing a nationwide exhaustive census data on births in South Korea.

## Methods

### Database

This study evaluated nationwide birth micro-data in South Korea across nine pre-pandemic years (2011–2019) and a pandemic year (2020) and examined whether the preterm birth rate, mean birthweight, and rates of low birthweight (LBW), macrosomia, small for gestational age (SGA), large for gestational age (LGA), and inappropriate birthweight have changed during the SARS-CoV-2 pandemic. The pre-pandemic period covered nine years (2011–2019) to reflect long-term trends in birth outcomes in South Korea^28^ regardless of the SARS-CoV-2 pandemic. Data of all births from 2011 to 2020 in South Korea were accessed via the Micro-data Access Service provided by Statistics Korea (KOSTAT)^28^. This study was approved by the Korea University Institutional Review Board (no. 2021GR0136) with a waiver of informed consent. All methods were performed in accordance with the relevant guidelines and regulations.

### Definition of birth outcomes

Preterm birth was defined as less than 37 weeks of gestation. LBW and macrosomia were defined as birthweights less than 2.5 kg and more than or equal to 4.0 kg, respectively. SGA and LGA births were identified using a previous study by Lee et al., which suggested sex- and gestational age-specific birthweight distributions^29^. Therefore, SGA and LGA births were those with birthweights less than the 10th percentile or more than or equal to the 90th percentile, respectively. Inappropriate birthweights were identified when there were one or more cases of LBW, macrosomia, SGA, or LGA. Only singleton births were analyzed.

### Statistical analysis

Two-sample tests for equality of proportion with continuity correction were performed to compare the rates of preterm birth, LBW, macrosomia, SGA, LGA, and inappropriate birthweights, and Welch’s two-sample t-test was performed for birthweight as a continuous outcome. Generalized linear models for predicting birth outcomes—including preterm birth, birthweight, LBW, macrosomia, SGA, LGA, and inappropriate birthweight with correspondence to the pre-pandemic or pandemic period—were built in three modes: not adjusting, adjusting for the long-term linear trend estimated by the pre-pandemic period, and adjusting for parental age, education, and marital status; sex parity; months since January 2011; and gestational age. Maternal and paternal education was dichotomized into “college education or higher” and “less than a college education.” Parity was dichotomized into “first birth” and “second birth or more.” Multi-collinearity was checked for all models by calculating variance inflation factors. To test for the trends of birth outcomes throughout the study period, chi-squared test for trend in proportions and Pearson’s product-moment test were performed. R statistics software version 4.1.2 (R Foundation for Statistical Computing, Vienna, Austria)^30^ and R packages including car^31^, ggplot2^32^, readr^33^, and stats^30^ were used for statistical analysis.

## Results

There were 3,481,423 singleton births in the pre-pandemic period (from January 2011 to December 2019), and 255,024 deliveries occurred during the pandemic period (from January to December 2020). The frequencies and proportions of preterm, LBW, macrosomia, SGA, LGA, inappropriate birthweight deliveries, and birthweight in the pre-pandemic and pandemic periods are shown in Table 1. Odds Ratios (OR) or coefficients for unadjusted, trend-adjusted, and all-adjusted models and their 95% Confidence Intervals (CI) are in Table 2.

**Table 1:** Differences in birth outcomes between pre-pandemic and pandemic period.

Variables	Pre-pandemic (Jan. 2011–Dec. 2019)	Pandemic (Jan.–Dec. 2020)	P-value*
Singleton births	3,481,423	255,024	–
Preterm births	168,361 (4.8%)	13,995 (5.5%)	< 0.001
Birthweight (kg)	3.23 ± 0.43	3.21 ± 0.43	< 0.001
LBW^†^ (< 2.5 kg)	129,807 (3.7%)	10,242 (4.0%)	< 0.001
Macrosomia (≥ 4.0 kg)	115,294 (3.3%)	6997 (2.7%)	< 0.001
SGA^‡^	223,972 (6.4%)	14,292 (5.6%)	< 0.001
LGA^§^	385,570 (11.1%)	28,082 (11.0%)	0.342
Inappropriate birthweight	672,167 (19.3%)	47,600 (18.7%)	< 0.001
Maternal age (years)	32 ± 4	33 ± 4	< 0.001
Paternal age (years)	34 ± 5	36 ± 5	< 0.001
Gestational age (weeks)	39 ± 2	38 ± 1	< 0.001
Maternal higher education	2,601,642 (75%)	200,896 (79%)	< 0.001
Paternal higher education	2,574,320 (74%)	193,460 (76%)	< 0.001
Sex (male)	1,788,175 (51.4%)	130,502 (51.2%)	0.063
Birth out of marital status	40,501 (1.2%)	4228 (1.7%)	< 0.001
Parity (1)	1,838,400 (53%)	146,350 (57%)	< 0.001

Frequency, proportion, and means of deliveries with birth outcomes and covariates used in the multivariable generalized linear regressions in the pre-pandemic (January 2011–December 2019) and SARS-CoV-2 pandemic (January 2020–December 2020) periods are shown.

*P-values from a two-sample test for equality of proportion with continuity correction for preterm birth, underweight birth, overweight birth, SGA, LGA, and inappropriate birthweight. A P-value from Welch’s two-sample t-test for birthweight.

^†^LBW: low birthweight, < 2.5 kg.

^‡^SGA: small for gestational age.

^§^LGA: large for gestational age.

**Table 2:** Odds ratios or coefficients of adverse birth outcomes in SARS-CoV-2 pandemic period compared to pre-pandemic period.

Birth outcomes	OR* (95% CI^†^) or coefficient		
	Unadjusted model	Trend-adjusted model	All-adjusted model
Preterm births	1.14 (1.12–1.16)	0.993 (0.987–0.991)	0.968 (0.948–0.988)
Birthweight (kg)	− 2.12 × 10^−2^ (− 2.30 × 10^−2^ to − 1.95 × 10^–2^)	− 1.11 × 10^−2^ (− 1.31 × 10^−2^ to − 9.10 × 10^–3^)	− 1.27 × 10^−3^ (− 2.95 × 10^−3^ to − 4.17 × 10^−4^)
LBW^‡^ (< 2.5 kg)	1.08 (1.06–1.10)	1.00 (0.980–1.03)	0.955 (0.928–0.984)
Macrosomia (≥ 4.0 kg)	0.824 (0.804–0.844)	0.913 (0.888–0.938)	0.923 (0.898–0.949)
SGA^§^	0.863 (0.849–0.879)	0.995 (0.975–1.01)	0.975 (0.955–0.994)
LGA^¶^	0.994 (0.981–1.01)	0.959 (0.945–0.973)	0.952 (0.938–0.966)
Inappropriate birthweight	0.959 (0.949–0.969)	0.974 (0.963–0.986)	0.946 (0.934–0.957)

Odds ratios or coefficients estimated from model 1: the unadjusted univariable model; model 2: trend-adjusted; and model 3: adjusted for for parental age, gestation age, parental education level, marital status of parents, parity, and months from January 2011; are shown.

*OR: odds ratio.

^†^CI: confidence interval.

^‡^LBW: low birthweight, < 2.5 kg.

^§^SGA: small for gestational age.

^¶^LGA: large for gestational age.

A two-sample test for equality of proportion with continuity correction for preterm birth rate (Table 1) and an unadjusted univariate generalized linear model (Table 2) showed that there was a significant increase in preterm births during the pandemic. However, after adjusting for maternal and paternal age, gestational age, maternal and paternal education level, marital status, sex, parity, and a long-term trend throughout the study period, a significant decrease in the proportion of preterm births (OR: 0.968; 95% CI 0.948–0.988) was observed. Welch’s two-sample t-test for the mean birthweight in the pre-pandemic and pandemic periods revealed a significant birthweight decline during the pandemic. The adjusted multivariable generalized linear model estimated a 1.27 g decline in birthweight during the pandemic period compared to the pre-pandemic period. LBW, macrosomia, SGA, and inappropriate birthweight rates were found to decrease during the pandemic period, but a proportion test showed that LGA rates remained similar. In Table 1, Maternal and paternal age significantly increased, while gestational age decreased. There were also increases in parental fulfillment of higher education, deliveries outside of marriage, and primiparous mothers between the pre-pandemic and pandemic periods.

**Figure 1:**
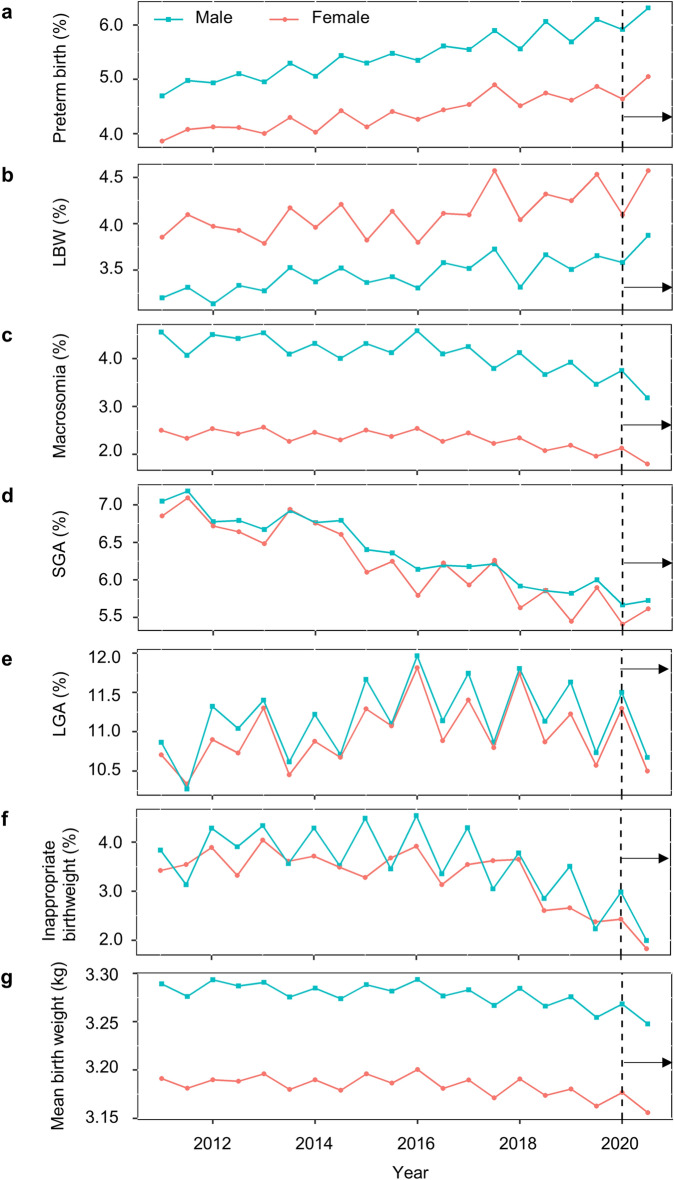
Long-term trends of inappropriate birth outcomes in South Korea. Rates of preterm births (**a**), Low Birthweight, LBW (**b**), Macrosomia (**c**), Small for Gestation Age, SGA (**d**), Large for Gestation Age, LGA (**e**), Inappropriate birthweights (**f**), and Mean birthweights (**g**) from 2011 to 2020. Females and males are represented in red circles and blue squares, respectively. In chi-squared tests for trend in proportions, all rates (**a**–**f**) for both sex had increasing (**a**, **b**, **e**) and decreasing (**c**, **d**, **f**) trends with p < 0.001 from 2011 to 2020. Mean birthweights (**g**) had decreasing trend with p = 0.005 for males and p = 0.002 for females by Pearson’s product-moment test. The SARS-CoV-2 pandemic started at the beginning of 2020, indicated by dashed black vertical lines and solid arrows.

Figure 1 shows the rates of preterm birth, LBW, macrosomia, SGA, LGA, inappropriate birthweight, and mean birthweight from 2011 to 2020. All rates for both males and females had increasing (preterm birth rate, LBW rate, and LGA rate) or decreasing (macrosomia rate, SGA rate, and inappropriate birthweight rate) trends with p < 0.001. Mean birthweight of both sex had decreasing trend with p = 0.005 in males and p = 0.002 in females. The mean birthweights of neonates born at 37, 38, and 39 weeks of gestation are shown in Supplementary Fig. S1.

## Discussion

To the researchers’ knowledge, this is the largest and most comprehensive nationwide study to date on the impact of the SARS-CoV-2 pandemic on preterm birth and birthweight. The current study found that the SARS-CoV-2 pandemic was negatively associated with preterm birth, LBW, macrosomia, SGA, LGA, and inappropriate birthweights.

We found long-term trends in birth outcomes throughout the study period independent of the SARS-CoV-2 pandemic and adjusted for the linear trends by including the months from January 2011 in the multivariable models.

Wagner et al. found that term infants born during the SARS-CoV-2 pandemic period had a significantly higher birthweight^21^, and Li et al. reported that the birthweight of newborns born after the Chinese government's strict lockdown in Wuhan starting on January 23, 2020, was significantly heavier than before lockdown when considering full-term or close-to-full-term births^27^. Sun et al. identified pregnant women from January 1, 2019, to December 31, 2020, using medical claims data and noted that from March 1 to December 31, 2020, the SARS-CoV-2 pandemic period was associated with a statistically significant higher risk of poor fetal growth (RR: 1.07; 95% CI 1.03–1.11)^10^.

Several explanations may be considered regarding the differing effects of the pandemic on birthweight between studies. After the first SARS-CoV-2 case confirmed case on January 20, 2020, by February 2020, Korea had the second most reported SARS-CoV-2 cases in the world outside of China, with cumulative infection cases in the thousands^34^. However, the SARS-CoV-2 infection rate patterns in Korea started to change in March and remained flattened and stable by July 2020. Li et al. suggested that possible underlying contributory factors for larger birthweights might include food and nutrition changes due to market closure and lack of exercise after lockdown^27^. In Korea, no market closure or lockdowns that could affect the lifestyle, physical activity, and healthcare access of pregnant women in 2020 were imposed by the government. Indeed, in the result summary presentation of the Korean National Health and Nutrition Examination Survey 2020^35^, the aerobic physical activity practice rate in women did not change much from 42.7% in 2019 to 43.0% in 2020. Therefore, the adverse impact of the pandemic on birthweight in Korea in 2020 may have been limited.

Preventive measures, such as social distancing, wearing face masks, and hand hygiene, have been enforced in South Korea; as such, mothers’ personal hygiene may have improved, and infectious diseases other than SARS-CoV-2 may also have been suppressed during the pandemic period. However, further studies are required to determine the effect of enhanced hygiene on fetal growth and to evaluate whether the impact of the pandemic on pregnant women differed by nation, depending on the SARS-CoV-2 incidence in each country and the respective government’s policies to prevent the rapid spread of the disease.

The impact of the pandemic may vary depending on the time of pregnancy. Several studies suggest that maternal psychological stress may be associated with an increased risk of low birthweight^22–25^. In particular, maternal exposure to severely stressful life events—especially in the first trimester, but not in the second and third trimesters—has been known to have a greater effect on birthweight, suggesting that the early trimester of pregnancy is crucial in terms of the impact of stressful life events on fetal growth^22^. At the end of December 2019, a cluster of cases of SARS-CoV-2 was first reported in Wuhan, China^36^; on January 30, 2020, the WHO declared SARS-CoV-2 a “public health emergency of international concern”. Therefore, the SARS-CoV-2 pandemic may have had little impact on the birthweight of newborns born in early 2020. However, in an ad hoc study re-defining the pandemic period as being from July to December 2020 and the pre-pandemic period as being the same months in 2011–2019, the ORs in all-adjusted models in Supplementary Table S1 were qualitatively similar to those in Table 2, except for that the 95% CIs of adjusted ORs for preterm birth rate expanded to include 1.00. Significance of ORs for LBW, macrosomia, SGA, LGA, and inappropriate birthweight rates remained significantly and negatively associated in the ad hoc study.

In Fig. 1, long-term trends were observed that increasing preterm birth rate and LBW rate, decreasing macrosomia, SGA, inappropriate birthweight rates, and mean birth weight over time, from years earlier than the SARS-CoV-2 pandemic started. The preterm birth rate is in increasing trend in recent decades worldwide^37,38^ and South Korea is no exception^28^. Increasing preterm birth rates are affected by multiple factors, including an increasing proportion of pregnant women over 35 years old, and increasing number of multiple births resulting from greater use of assisted reproduction technology^39,40^. Because we analyzed singleton births only, a rise in maternal age resulting in shorter gestation age may have played a major role in the incline of preterm birth rate. Despite the increase in LBW rate, SGA rate have declining trend which may seem paradoxical. It could be explained by the shortening of the gestation age. Indeed, in Fig. S1 neonates with the same gestation age have little deviation in birthweight from year to year. Table 2 shows the importance of adjusting for those long-term trends. For example, preterm birth rate seemed to be increased in pandemic period compared with in pre-pandemic period without adjustment but adjusting for the long-term trend regardless of the pandemic indicated a decrease in the pandemic period.

The effect of the SARS-CoV-2 pandemic on preterm birth is inconsistent across previous reports. Some studies report a decreased risk of preterm birth^5–8^, while others reported that they found no evidence of changes^10–12^. Evidence supporting a decreased risk of preterm births during the pandemic period was provided by the current study.

Some limitations should be considered when interpreting the current findings. Some clinical information—such as maternal BMI, height, body weight, and post history—that may affect birthweight^41^ and information on subtypes of preterm births was lacking. The national birth micro-data also does not contain information on the history of SARS-CoV-2 infection during pregnancy. Therefore, the effect of SARS-CoV-2 infection on birthweight cannot be completely excluded. However, there will likely be little effect from these infected women, because the number of infected mothers in 2020 was as small as 700^42^. Despite these limitations, one key strength of this study is its large, nationwide coverage.

Declines in the rates of preterm birth, LBW, macrosomia, SGA, LGA, and inappropriate birthweight were observed during the pandemic (2020) compared with the pre-pandemic period (2011–2019), after adjusting for parental age, education level, marital status, parity, gestational age, and long-term trends. Further studies are required to understand this phenomenon.

## Supplementary Information

Supplementary Information.

## Data Availability

Data of all births from 2011 to 2020 in South Korea were accessed via the Micro-data Access Service provided by Statistics Korea (KOSTAT) on the following web page: https://mdis.kostat.go.kr/.
