# Supplementary material for: Changes in preterm birth and birthweight during the SARS-CoV-2 pandemic: a nationwide study in South Korea
Source: Sci Rep. 2022 Sep 29;12:16288. doi: 10.1038/s41598-022-20049-2 (PMC9520997; doi:10.1038/s41598-022-20049-2)
Supplement: Supplementary file 1 — Supplementary Information. [file 41598_2022_20049_MOESM1_ESM.pdf]

Changes in Preterm Birth and Birthweight During the SARS-CoV-2 Pandemic: A Nationwide Study in South Korea

Jeongeun HWANG, PhD[1,2]; Seokjoo Moon, MS[3], Kyu-Dong CHO, PhD[4]; Min-Jeong OH, MD[5]; Geum Joon CHO, MD[5*]

[1]Division of Medical Oncology, Department of Internal Medicine, Korea University College of Medicine, Seoul, Republic of Korea

[2]Department of Biomedical Research Center, Korea University Guro Hospital, Seoul, Republic of Korea

[3]Smart Healthcare Center, Korea University Guro Hospital, Seoul, Republic of Korea

[4]Big Data Department, National Health Insurance Service, Gangwon-do, Korea

[5]Department of Obstetrics and Gynecology, Korea University College of Medicine, Seoul, Republic of Korea

Supplementary Information

Figure S1. Mean birthweights of neonates at 37 (**a**), 38 (**b**), and 39 (**c**) weeks of gestation over time in South Korea.

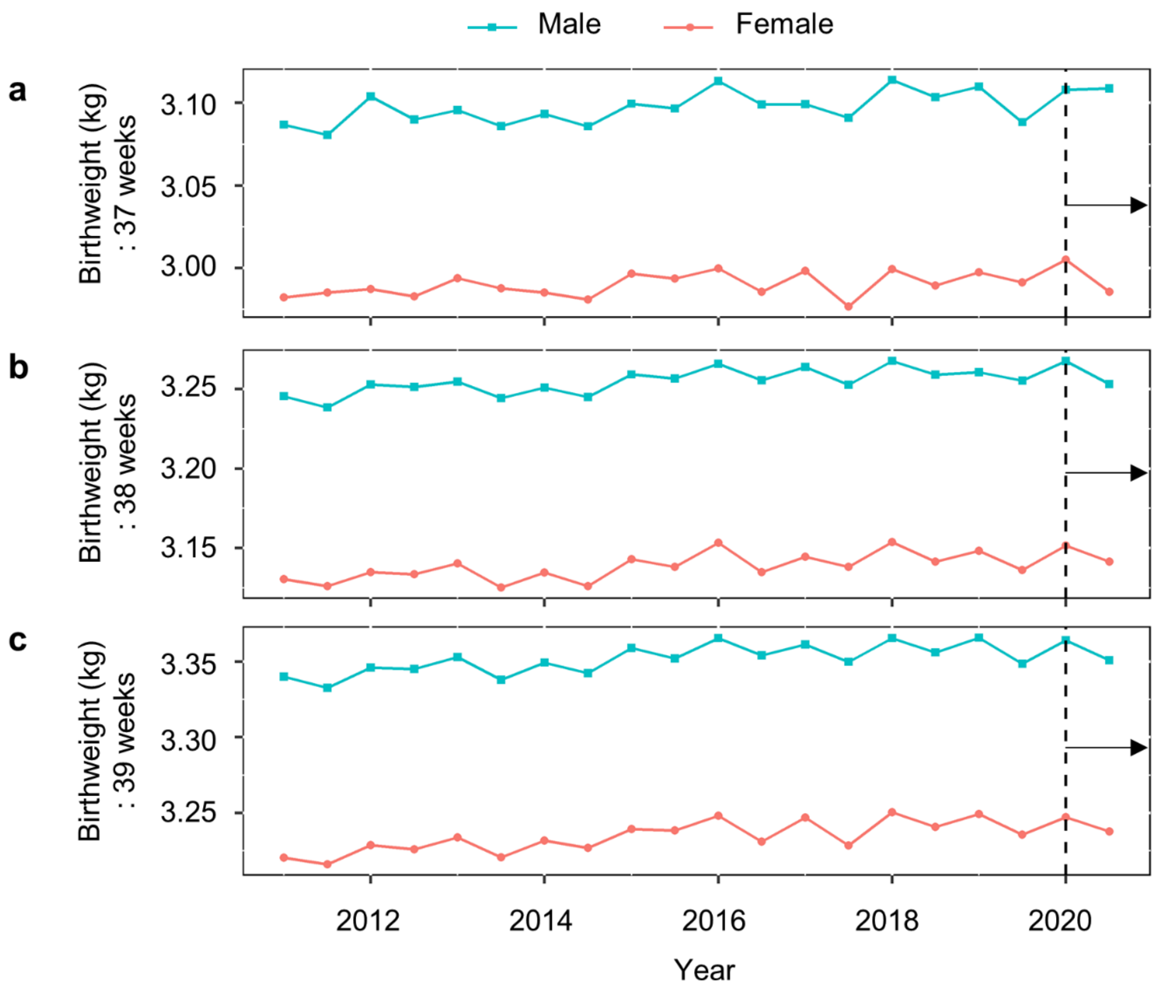

Table S1.

Table S1. Odds ratios or coefficients of adverse birth outcomes in SARS-CoV-2 pandemic period (July–December, 2020; 122,193 singleton births) compared to pre-pandemic period (July–December, 2011–2019; 1,672,465 singleton births). Odds ratios or coefficients estimated from model 1: the unadjusted univariable model; model 2: trend-adjusted; and model 3: adjusted for for parental age, gestation age, parental education level, marital status of parents, parity, and months from January 2011; are shown.

Birth outcomes	OR* (95% CI†) or coefficient		
	Unadjusted model	Trend-adjusted model	All-adjusted model
Preterm births	1.15 (1.13–1.18)	1.00 (0.972–1.03)	0.976 (0.948–1.01)
Birthweight (kg)	–0.025 (–0.028—0.023)	–0. 013 (–0.016—0.01)	–0. 002 (–0.004–0.001)
LBW‡ (< 2.5 kg)	1.10 (1.07–1.13)	1.02 (0.983–1.05)	0.963 (0.924–1.00)
Macrosomia (≥ 4.0 kg)	0.787 (0.758–0.816)	0.877 (0.841–0.914)	0.890 (0.854–0.928)
SGA§	0.864 (0.842–0.886)	0.991 (0.963–1.02)	0.972 (0.944–1.00)
LGA¶	0.981 (0.963–1.00)	0.954 (0.934–0.975)	0.947 (0.926–0.967)
Inappropriate birthweight	0.955 (0.941–0.970)	0.974 (0.957–0.990)	0.943 (0.927–0.960)

*OR: Odds Ratio

†CI: Confidence Interval

‡ LBW: Low Birthweight, < 2.5 kg

§ SGA: Small for Gestational Age

¶LGA: Large for Gestational Age
